# Ultra-flexible nonvolatile memory based on donor-acceptor diketopyrrolopyrrole polymer blends

**DOI:** 10.1038/srep10683

**Published:** 2015-06-01

**Authors:** Ye Zhou, Su-Ting Han, Yan Yan, Li Zhou, Long-Biao Huang, Jiaqing Zhuang, Prashant Sonar, V. A. L. Roy

**Affiliations:** 1 Department of Physics and Materials Science City University of Hong Kong, Tat Chee Avenue, Kowloon Tong, Hong Kong SAR; 2 School of Chemistry, Physics and Mechanical Engineering, Queensland University of Technology (QUT), GPO Box 2434, Brisbane, QLD 4001, Australia

## Abstract

Flexible memory cell array based on high mobility donor-acceptor diketopyrrolopyrrole polymer has been demonstrated. The memory cell exhibits low read voltage, high cell-to-cell uniformity and good mechanical flexibility, and has reliable retention and endurance memory performance. The electrical properties of the memory devices are systematically investigated and modeled. Our results suggest that the polymer blends provide an important step towards high-density flexible nonvolatile memory devices.

Light weight, mechanically flexible, wearable and stretchable electronic devices are emerging research areas for modern electronics^1^,^2^,^3^,^4^,^5^. Organic bistable memory devices are highly promising technologies for future ultrahigh density data storages owing to low cost, simple structure, mechanical elasticity and facile processing^6^,^7^,^8^. To date, various insulating or semiconducting materials including carbon nano-materials^9^, small molecules^10^,^11^,^12^,^13^, chalcogenides^14^,^15^, conjugated or non-conjugated polymers^16^,^17^,^18^,^19^,^20^,^21^, transition metal oxides^22^,^23^,^24^,^25^, organic composites^26^,^27^ and organic/inorganic hybrid materials^28^,^29^,^30^ have been adopted for resistive switching memories. In these devices, the data information is stored on the basis of response to high or low conductivity for applied voltages. Polymers have attracted a great amount of attention as future memory elements due to the high-throughput, easy solution processability, printability, good scalability and three-dimensional stackability^31^,^32^,^33^. Metal nanomaterials^34^,^35^,^36^,^37^, metal oxide nanoparticles^38^, small molecules^39^, carbon-based materials^3^,^40^,^41^,^42^ and metal chalcogenides^43^ have been inserted as charge acceptors/fillers in polymer-based memories. However, most of these filler materials require elaborate and expensive processing technology thereby impeding the large-scale fabrications. The reproducibility and stability of these devices also need to be further improved due to the aggregation of nano-components and irregular phase separation^44^. Therefore, developing fully polymer based nonvolatile memories with the following capability is of great demand: i) Controlled surface morphologies and electrical properties; ii) Easy fabrication and large area printability; iii) Facile tailoring of the molecular structures of desired materials; iv) Highly mechanical flexibility and stable memory behavior.

Based on the above considerations, here we introduce a nonvolatile ultra-flexible memory array with fully polymer based active layer constructed on a 12-μm-thin polyethylene naphthalate (PEN) substrate. Diketopyrrolopyrrole-naphthalene copolymer (DPP-TNT) is mixed with polymethyl methacrylate (PMMA) as the functional material in the memory device via a facile solution processed approach. DPP based donor-accepter semiconducting polymer is highly promising for thin-film transistors and photovoltaics due to facile tailoring of the molecular structures^45^,^46^. The electron-deficient DPP moiety can induce large intramolecular charge transfer due to the strong electron-withdrawing fused lactam rings and the copolymers based on DPP show high ambipolar or unipolar semiconducting performance^47^. However, few reports on the electrical bistable properties of DPP based polymer can be found until now. The DPP-TNT with relative high mobility acts as the charge trapping center and carrier transporting medium, whereas the PMMA serves as the blocking matrix of the charge carriers. The device exhibits a low operation voltage, a high on/off ratio of 10^4^, high cell-to-cell uniformity and a retention time of longer than 10^5^ s. The electrical properties of the as-fabricated devices are systematically investigated and modeled. The blended polymers composites exhibit excellent stability and flexibility in data storage applications. The newly designed DPP-based polymer hybrid approach is quite beneficial for low-cost and large scale fabrication of memory devices and can create novel approaches for data storage technology in applications such as portable electronics, rollable displays and electronic artificial skin.

## Results

### Device structure

**Figure 1:**
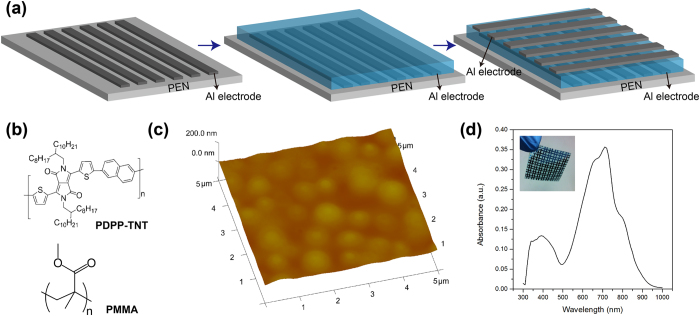
(**a**) Illustration of the fabrication of the ultra-flexible memory device. (**b**) The chemical structures of PMMA and DPP-TNT. (**c**) Tapping mode AFM image of the PMMA:DPP-TNT film. (**d**) UV–visible spectrum of PMMA:DPP-TNT film. Inset: Optical image of the fabricated ultra-flexible memory.

The fabrication procedure of the ultra-flexible memory device is shown in Fig. 1a. Al electrodes were firstly deposited on the PEN substrate with defined patterns. DPP-TNT was synthesized according to our previous work^48^. PMMA (10 mg/ml) and DPP-TNT (5 mg/ml) were dissolved in chlorobenzene and then spin-coated on a pre-patterned PEN substrate. Other concentrations of DPP-TNT in PMMA have also been investigated and 5 mg/ml was found to be the optimized ratio with stable and repeatable device performance. Finally, top Al electrodes were coated on top of the polymer layer. The memory cell has an active area of 1 × 1 mm^2^, as dictated by the shadow mask used for the electrode deposition. The chemical structures of PMMA and DPP-TNT used in the active layer are shown in Fig. 1b. The morphologies of the PMMA:DPP-TNT film were characterized by atomic force microscope (AFM) and depicted in Fig. 1c. The image clearly demonstrates the formation of distinct polymer aggregation after thermal treatment, as a result of the polarity difference between DPP-TNT and PMMA. The existence of the DPP-TNT in PMMA matrix is also confirmed by the UV-visible absorption spectra as shown in Fig. 1d. The spin-coated film shows two broad absorption bands in the 300-500 nm and 500-800 nm regions, which is attributed to the absorption of solid state DPP-TNT^48^. The photograph of the as-fabricated 14 × 14 devices is shown in the inset of Fig. 1d.

### Electrical characteristics

**Figure 2:**
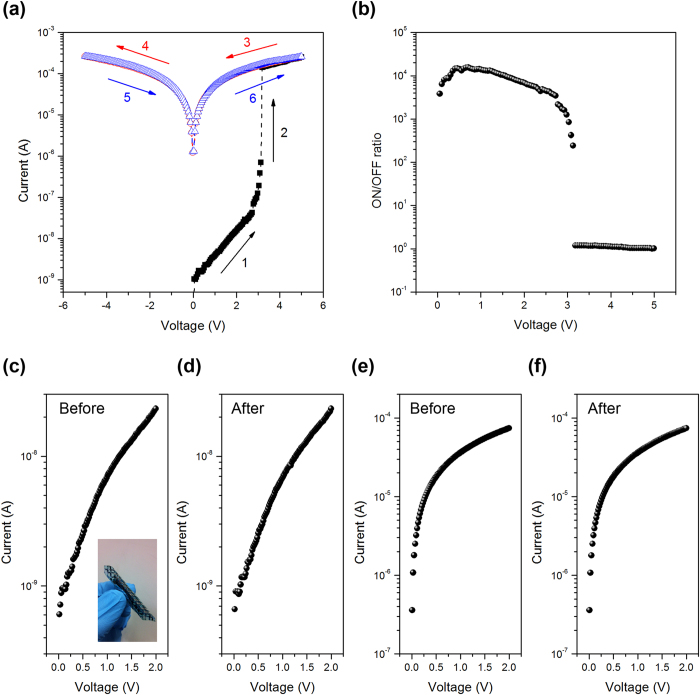
(**a**) The electrical characteristics of the ultra-flexible memory cells. (**b**) ON/OFF ratio of the memory device. (**c**) The OFF state of the memory cell before rolling. (**d**) The OFF state of the memory cell after rolling. (**e**) The ON state of the memory cell before rolling. (**f**) The ON state of the memory cell after rolling.

Figure 2a shows the typical memory characteristics of the ultra-flexible memory cells. The arrows in the figures reveal the sweeping direction of the applied voltage. Initially the polymer memory was in a high resistance state and we denoted this state as OFF state. The current started in a low conductivity level and increased progressively after applying the positive bias. When the voltage which applied in the electrodes exceeded the switch voltage of 2.7 V, the device transferred to a low resistance state. A set process is happed in this period, and this low switch voltage suggests the fabricated polymer memory can work with low energy consumption. Meanwhile, the high ON/OFF ratio as shown in Fig. 2b promises a low probability of misreading due to the well separated ON and OFF state during the operation. The subsequent scan from 5 V to -5 V (sweep 3 and 4) did not change the device from low resistance state to high resistance state, and the memory cell kept its low resistance state during the subsequent scan form -5 V to 5 V (sweep 5 and 6). After the transition, the OFF state cannot be obtained by utilizing a reverse voltage sweep or even turning off the power, indicating our polymer memory cell is a write-once-read-many-times (WORM) type memory^49^,^50^. The polymer WORM memory is very advantageous for the data storage with ultralow cost and persistent usage, eliminating the demand of expensive, bulky and slow mechanical drives used in conventional optical and magnetic memories^51^,^52^. In addition, we did not observe the electrical bistable phenomenon in the PMMA device, indicating the important role of DPP-TNT in the nonvolatile memory effect. The flexible arrays were electrically characterized before and after being physically rolled. Figure 2c–2f illustrate the electrical characteristics of the polymer memory device at ON and OFF state. During the test, severe rolling of the substrate at the curvature radii of 3 mm did not degrade the memory performance, suggesting the excellent flexibility of the device. The electrical performances of the memory devices as a function of bending cycles are shown in supporting information (Supplementary Figure 1). The memory did not show significant degradation of ON/OFF ratio for 500 cycles. The memory properties could be reproducible after repetitive rolling the PEN substrates, demonstrating that the electrical performance of the polymer belnds is stable no matter their physical presentation^53^. Further enhancement of the flexibility can be realized by using flexible electrode materials such as graphene^49^.

### Conduction mechanism

**Figure 3:**
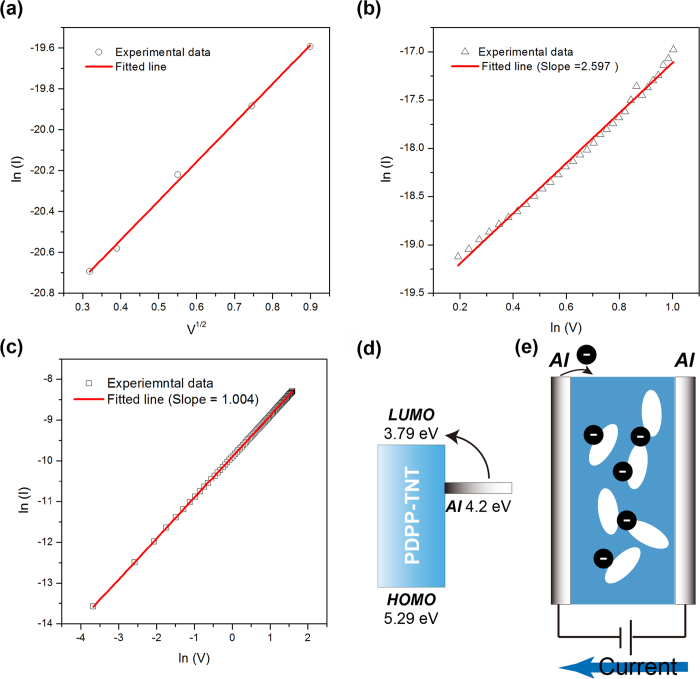
(**a**) Experimental data and fitted lines of the I-V characteristics with TE model. (**b**) Experimental data and fitted lines of the I-V characteristics with SCLC model. (**c**) Experimental data and fitted lines of the I-V characteristics with Ohmic current model. (**d**) Energy diagram of the memory device. (**e**) Proposed operating mechanism of the memory device.

In order to investigate the charge transport and conduction mechanism in the polymer blends, the experimental current-voltage (I-V) curve were fitted by different carrier transport models^54^ as shown in Fig. 3a–3c. The I-V curves were obtained by sweeping with a forward bias from 0 V to 1 V and the experimental data could be fitted well with equation 1 by using the thermionic emission (TE) process.
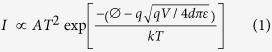
where I is the current, V is the applied voltage, A is the Richardson constant, T is the temperature, k is the Boltzmann constant, 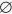 is the Schottky energy barrier, q is the electron charge, d is the film thickness and ε is the electrical constant. This suggests that the charge injection through the energy barrier between Al and PMMA dominates the conduction mechanism. As the voltage exceeding 1 V, the I-V curves could be fitted with equation 2 by using a space-charge-limited current (SCLC) process.
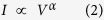
where I is the current, V is the applied voltage. The results indicate that the captured carriers in DPP-TNT can act as space charges^55^. In this process, the current through the polymer device changes from a charge injection current to a predominantly space-charge-limited current.

The ON state current can be fitted well with equation 3 by an Ohmic conduction model.
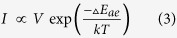
where I is the current, V is the applied voltage, ΔE_ae_ is the activation energy of electron, k is the Boltzmann constant and T is the temperature. The obtained slop is nearly equal to 1, and we can propose that the charge carrier transport process is dominated by the Ohmic conduction when the device is in the ON state.

To investigate the observed memory characteristics, we considered the highest occupied molecular orbital (HOMO) and lowest unoccupied molecular orbital (LUMO) of DPP-TNT and the work function of Al electrodes as shown in Fig. 3d. The energy barrier between the work function of Al and LUMO is 0.41 eV, which is smaller than that (1.09 eV) of the work function of Al with the HOMO. Thus electron injection is more favorable and dominates the conduction process in the memory device. On the basis of the aforementioned results, the operating mechanism of the memory device is proposed and depicted in Fig. 3f. When the positive forward voltage is applied to the Al electrode, the electrons emitted from the Al electrode are injected into the PMMA. The high resistance state current in low voltage can be attributed to the TE process and that in the high voltage is related to the SCLC process with the accumulation of space charges in the PMMA layer. The trapping sites arise from the DPP-TNT polymer chain, which is composed of DPP acceptor and naphthalene donor blocks. The electron-donating and with-drawing groups can function as charge trapping sites^31^, since the pure PMMA device does not exhibit such memory effect. When the applied voltage is large enough, many electrons injected from the Al electrode are captured by the DPP-TNT molecules, resulting in an increase in film conductivity. The ON state current which flow through the device is attributed to the Ohmic process^56^. After that, even though a large backward voltage is applied to the device, the captured electrons in DPP-TNT molecules cannot be emitted since the lowest unoccupied molecular orbital (LUMO) of DPP-TNT is much deeper than the LUMO of PMMA. The electrons are stabilized by the PMMA matrix, resulting in nonvolatile nature of the memory device.

### Device reliability

**Figure 4:**
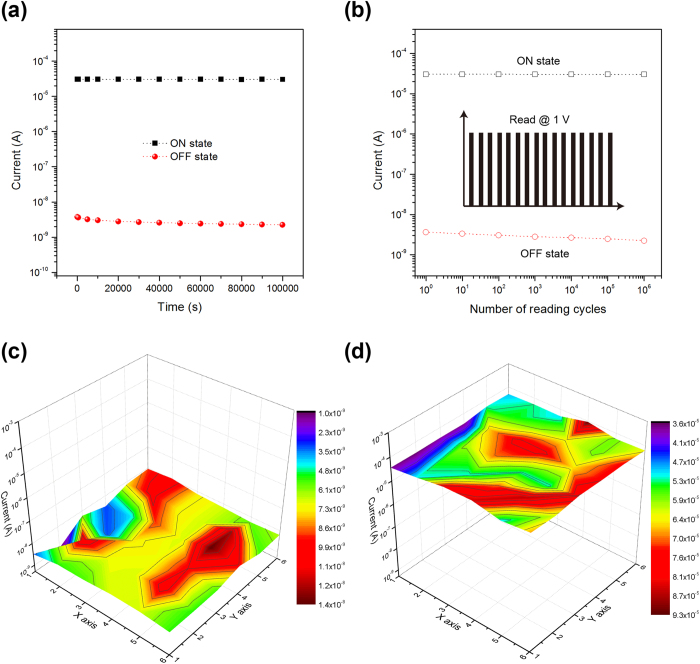
(**a**) Retention characteristics of the memory cell. (**b**) Reading cycle test of the memory cell. (**c**) The OFF currents of randomly selected 6 × 6 cells. (**d**) The ON currents of randomly selected 6 × 6 cells.

In addition to the low power consumption and low misreading probability, the reliability of the flexible memory device is an important property for practical memory applications. Data retention test were conducted before and after writing the memory cell as shown in Fig. 4a. No significant degradation of the conductivity for both ON and OFF states was observed after considerable duration of 10^5^  s under a constant 1 V read voltage, demonstrating the excellent stability of the memory cell, which is comparable with other reports^41^,^57^. Fig. 4b shows the pulse reading stress characteristics of the memory device. The device was read with pulse reading bias of 1 V for 1 ms continuously as illustrated in the inset. The ON and OFF states were stable after 10^6^ continuous reading pulses. Such high air stability can ensure the reliable operation of the ultra-flexible memory device without particular encapsulation. Fig. 4c,d show the OFF and On currents of randomly selected 6 × 6 cells. Those memory cells exhibited similar ON and OFF current levels, which suggest the good uniformity of the memory array. The results also reveal that the electrical characteristics are repeatable with good accuracy in the memory cell array.

## Discussion

An ultra-flexible polymer memory cell array based on conjugated copolymer DPP-TNT has been successfully fabricated through a simple, low temperature and cost-effective approach. An ON-OFF current ratio of around 10^4^ and a retention time of longer than 10^5^ s were achieved with good mechanical flexibility and air stability. The proposed strategy is the first report demonstrating DPP based donor-accepter polymer in nonvolatile resistive memories. DPP-TNT is soluble donor-acceptor novel material which is soluble in most of the common organic solvents due to a long branched alkyl chain, 2-octyl-1-dodecyl substituted on the N-atom of the DPP, making it as a promising candidate for large area printed electronics. The synthesis of this polymer is straightforward, easy and can be scaled up. The DPP and naphthalene fused ring aromatic comonomers are favorable for better π–π stacking. In addition to that DPP-TNT exhibits lower HOMO values (compared to conventional semiconducting polymers such as P3HT), which is important for making stable electronic devices. For the above mentioned reasons, we select this DPP-TNT polymer that exhibits stable performance in memory devices and such a multifunctional material can be used for various organic electronic devices. In addition, a donor-acceptor polymer with high solubility and promising performance can pave a way for large area flexible and printed electronics. Furthermore, we have demonstrated direct fabrication of nonvolatile memory on ultrathin (12 μm) polymer substrates, which are light-weight and can minimize electronic waste. The technology described here are not limited by the substrate thickness, which can be further decreased to be applied in imperceptible electronics^58^.

## Methods

### Materials

Detailed synthetic procedures for the preparation of DPP-TNT have been published previously^48^. PMMA (average M_w _~ 996,000) were purchased from Aldrich. All the solvents were used without further purification. The PEN substrates were provided by DuPont Teijin Films China Limited.

### Device fabrication

60-nm-thick Al bottom electrodes were thermally evaporated on PEN substrate at a pressure of less than 5 × 10^−6^  mbar with a deposition rate of 0.2 nm s^−1^. The polymer solution composed of DPP-TNT and PMMA in cholorobenzene was spin-casted onto the Al patterned PEN substrate by a spin-coater. The polymer thin films were then annealed at 120 ^o^C for 1 h in the nitrogen environment. The thickness of the polymer layer was determined to be about 100 nm. Finally, the 60-nm-thick Al top electrodes were deposited and patterned by metal shadow mask. The electrical measurements were then carried out on memory cells of 1 mm × 1 mm in size.

### Characterization

The AFM images of the polymer films were scanned in tapping mode by atomic force microscope (AFM, Veeco Multimode V). The absorption spectrum of the polymer film was obtained using a Perkin Elmer Lambda 750 UV–visible near-infrared spectrophotometer with integrating sphere. The electrical characterization of the memory cells were carried out by a Keithley 2612 source meter and an Agilent 4155c semiconductor parameter analyzer at room temperature in ambient atmosphere.

## Additional Information

**How to cite this article**: Zhou, Y. *et al.* Ultra-flexible nonvolatile memory based on donor-acceptor diketopyrrolopyrrole polymer blends. *Sci. Rep.* **5**, 10683; doi: 10.1038/srep10683 (2015).

## Supplementary Material

Supporting Information
