# Supplementary material for: Ultra-flexible nonvolatile memory based on donor-acceptor diketopyrrolopyrrole polymer blends
Source: Sci Rep. 2015 Jun 1;5:10683. doi: 10.1038/srep10683 (PMC4450595; doi:10.1038/srep10683)
Supplement: Supporting Information [file srep10683-s1.doc]

## Supporting Information

## Ultra-flexible nonvolatile memory based on donor-acceptor diketopyrrolopyrrole polymer blends

Ye Zhou^1^, Su-Ting Han^1^, Yan Yan^1^, Li Zhou^1^, Long-Biao Huang^1^, Jiaqing Zhuang^1^, Prashant Sonar^2^ and V. A. L. Roy^1^*

^1^Department of Physics and Materials Science and Center of Super-Diamond and Advanced Films (COSDAF), City University of Hong Kong, Hong Kong SAR

^2^School of Chemistry, Physics and Mechanical Engineering, Queensland University of Technology (QUT), GPO Box 2434, Brisbane, QLD 4001, Australia

Correspondence and requests for materials should be addressed to V. A. L. R. ([val.roy@cityu.edu.hk](mailto:val.roy@cityu.edu.hk))


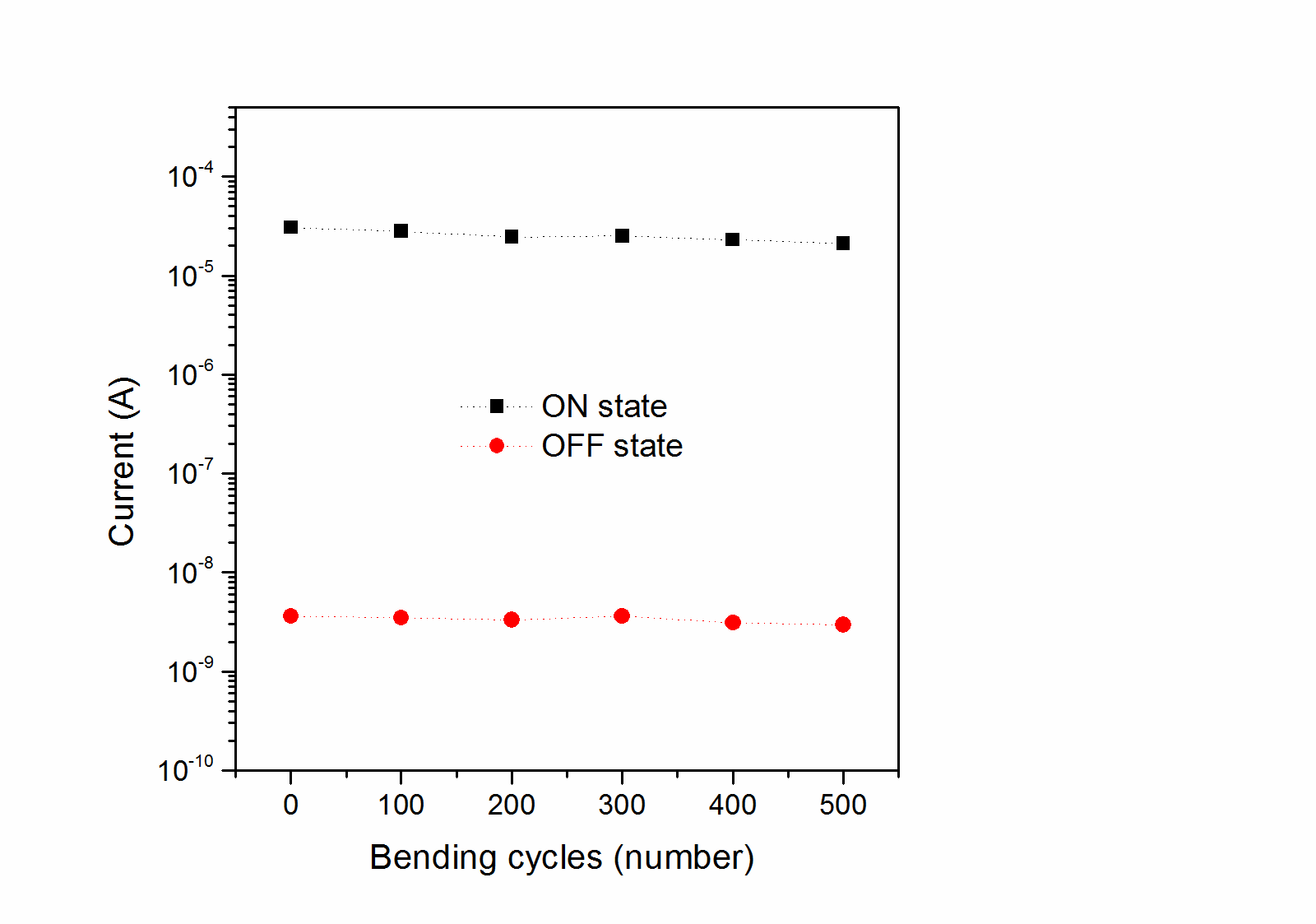


**Supplementary Figure 1.** The electrical properties of the memory devices with respect to the number of bending cycles.
